# Peripheral T Cell Non-Hodgkin's Lymphoma following Treatment of Hodgkin's Lymphoma

**DOI:** 10.1155/2015/438385

**Published:** 2015-01-13

**Authors:** Sun Hee Chang, Hye Ran Lee

**Affiliations:** ^1^Department of Pathology, Inje University Ilsan Paik Hospital, 170 Joohwa-ro, Ilsanseo-gu, Goyang 411-706, Republic of Korea; ^2^Department of Internal Medicine, Inje University Ilsan Paik Hospital, 170 Joohwa-ro, Ilsanseo-gu, Goyang 411-706, Republic of Korea

## Abstract

Previous reports have suggested that non-Hodgkin's lymphoma (NHL) is more likely to develop in patients with Hodgkin lymphoma (HL) compared to the general population. These two can occur synchronously or metachronously. We report here on a case of nodular sclerosis classical HL and T cell NHL that occurred in a patient metachronously. Peripheral T cell lymphoma (PTCL) of the patient was found about 2 years after treatment of classical HL. When the patient was diagnosed with HL, biopsy revealed typical RS cells, presenting positive for CD30 and CD15 and negative for CD79a and CD3 in immunohistochemistry. And PCR analysis showed IgH gene rearrangement; however, T cell receptor gene rearrangement and Epstein-Barr virus (EBV) were not detected on PCR analysis. After 2 years of treatment of HL, colonoscopic biopsy and lymph node biopsy showed CD3 positive atypical cells intermixed with small reactive lymphoid cells and plasma cells, indicating T cell lymphoma. PCR analysis demonstrated T cell receptor gene rearrangement and did not detect EBV. Although it is rare, synchronous or metachronous HL and NHL may occur. Therefore, we may need to ensure pathological confirmation, especially in case of lymphoma that did not respond to chemotherapy.

## 1. Introduction

It has been reported that the occurrence of Hodgkin's lymphoma (HL) and non-Hodgkin's lymphoma (NHL) in same patient is not rarer than expected [1]. A population based study found a greater than 3-fold increased incidence of NHL in patients previously given a diagnosis of HL [2]. A diffuse large B-cell lymphoma following previous nodular lymphocyte predominant HL can be seen the most common, but all types of NHL and HL have been observed, including T cell lymphoma (TCL) [3]. It can be developed synchronously and metachronously. Synchronous onset of HL and NHL at different anatomic sites or even both histologic manifestations present within the same tissue specimen referred to as composite lymphomas [1–3]. It is extremely rare and thought to be genetically identical with morphological differences due to different transformation events of common precursor cells [1]. Metachronously developed cases were considered to be attributed to complications of previous chemotherapy; however, they may be associated with immune system abnormalities [3]. Recent studies made progress to understand the cell of origin and clonality of RS cells. Studies showed that about 15% to 38% of RS cells expressed lymphoid antigens CD20 and 11% to 24% of these expressed CD3. Clonal immunoglobulin heavy chain gene rearrangements in RS cells were detected in 90–95% of HL cases. It was also revealed that 15% to 20% of RS cells expressing T cells antigens retain clonal* TCR* gene rearrangements [3–5].

We report a case of PTCL that seemed to develop metachronously in the patient with HL and review of literatures for histogenetic relationship between two lymphomas.

## 2. The Case Report

**Figure 1:**
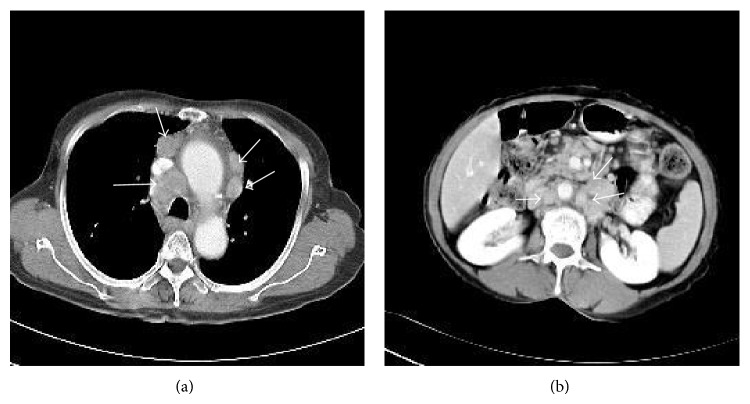
(a) A chest CT shows multiple enlarged lymph nodes in mediastinum. (b) An AP CT reveals multiple enlarged lymph nodes in abdominal retroperitoneal space. (White arrows: enlarged lymph nodes.)

**Figure 2:**
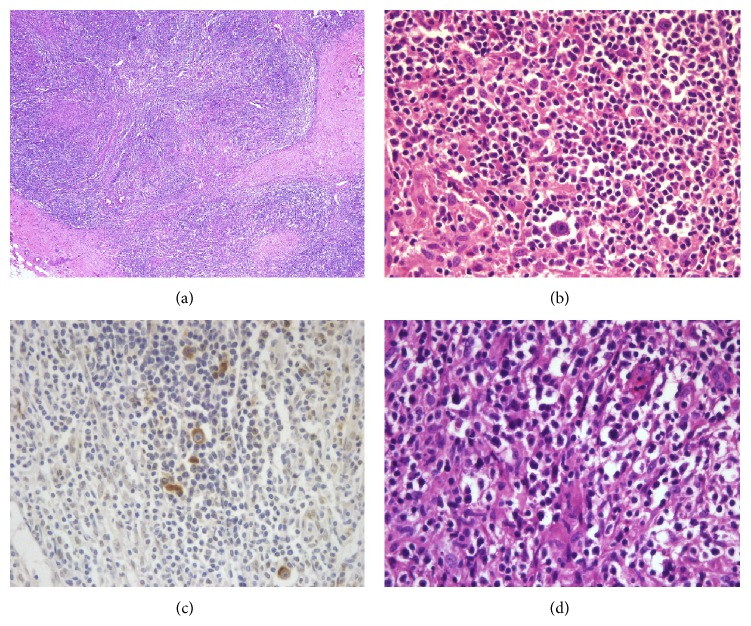
(a) The lymph node shows nodules separated by broad bands of fibrosis. (b) Reed-Sternberg cells and their variants are in the nodules. (c) Reed-Sternberg cells are CD30 positive. (d) Atypical lymphoid cells have hyperchromatic nuclei with discernible cytoplasm.

**Figure 3:**
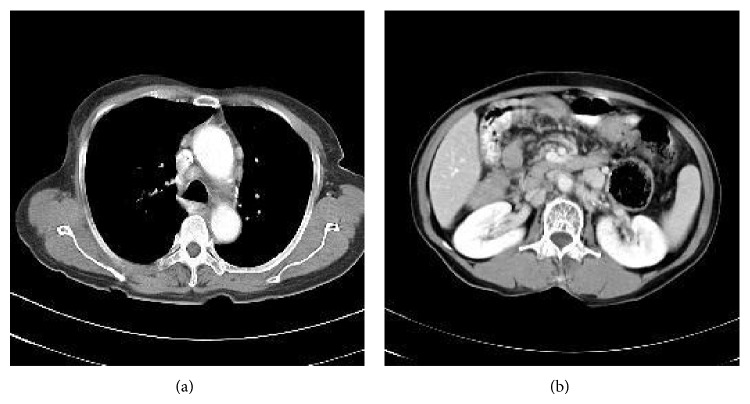
(a) A follow-up chest CT after 3 cycles of ABVD chemotherapy shows dramatic improvement of the lymphadenopathy in the mediastinum. (b) As compared with previous APCT, enlarged lymph nodes are decreased in number and size after 3 cycles of ABVD chemotherapy.

**Figure 4:**
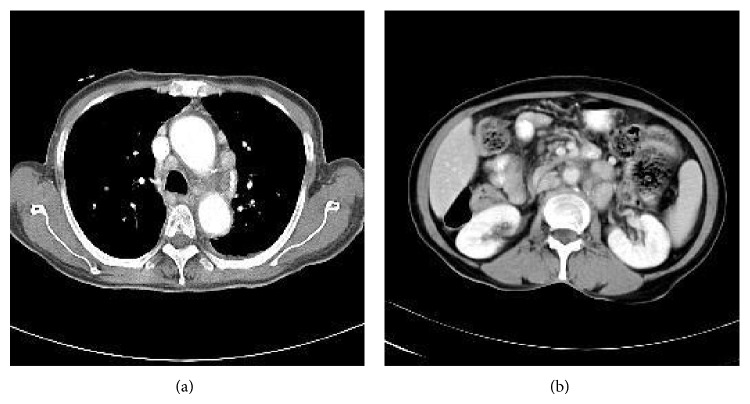
A chest CT (a) and an AP CT (b) show increased sizes of lymph nodes on mediastinum and retroperitoneal space after 12 months of follow-up loss.

A 64-year-old female patient came to the hospital complaining of abrupt weight loss of 10 kg in 2 months. She became 40 kg from 50 kg. She also complained of abdominal pain and bloating that has been annoying her for several months. She has been taking medicines for hypertension and diabetes. Multiple lymph nodes were palpable on right and left supraclavicular areas, right cervical area, and both inguinal areas. The largest one was measured 2.5 cm × 1.5 cm on left supraclavicular area. She had no history of fever and sweating. A neck computed tomography (CT) was performed and showed multiple lymphadenopathies on both supraclavicular and right jugular chains. A chest CT also showed multiple enlarged lymph nodes in mediastinum with small amount of pericardial effusion. An abdominal pelvis CT (AP CT) revealed enhanced multiple enlarged lymph nodes in abdominal retroperitoneal space (Figure 1). Excisional biopsy was performed on left supraclavicular lymph node and revealed Hodgkin's lymphoma (HL), nodular sclerosis type (NS) (Figure 2(a)). The biopsy revealed typical Reed-Sternberg (RS) cells and their variants (usually mononuclear cells and occasional lacunar cells) (Figure 2(b)). RS cells were immunohistochemically positive for CD30 (Figure 2(c)) and CD15 and negative for CD79a and CD3. Surrounding T cells were normal morphologically. PCR analysis showed IgH gene rearrangement. T cell receptor gene rearrangement was not detected. Epstein-Barr virus (EBV) was not detected on PCR analysis. Bone marrow examination showed no involvement of lymphoma. The echocardiography was performed to reevaluate pericardial effusion. It revealed that the ejection fraction was 60% and pericardial effusion was too small to be drained. Therefore, she was diagnosed with classical HL of nodular sclerosis type, stage IIIB, or possibly stage IV/BE due to pericardial involvement. According to International Prognostic Score (IPS) for risk stratification, she had 1 point by age of 64 years. Chemotherapy with doxorubicin, bleomycin, vinblastine, and dacarbazine (ABVD regimen) was administered. After 3 cycles of chemotherapy, a follow-up chest CT and an APCT were taken and showed partial response by RECIST criteria (Figure 3). However further chemotherapy was not given because she was intolerable to chemotherapy due to gastrointestinal (GI) symptoms including diarrhea and abdominal bloating. In order to find out the causes of GI symptoms, colonoscopy was performed and colonoscopic biopsy showed chronic nonspecific colitis with focal erosion on rectosigmoid colon. After 12 months of follow-up loss, CT showed slightly increased sizes of lymph nodes on neck, mediastinum, and retroperitoneal space (Figure 4). She was retreated again with 3 cycles of chemotherapy of ABVD regimen. Following APCT showed that size and numbers of multiple enlarged lymph nodes in retroperitoneal space and mesentery were decreased but persistently remained. It also showed diffuse edematous thickening of sigmoid colonic wall and rectal wall. Colonoscopic biopsy was reperformed and revealed malignant lymphoma of T cell phenotype with active ulcer along with necrosis on rectum and sigmoid colon, which was consistent with peripheral T cell lymphoma (TCL) involving rectum and sigmoid colon. In order to confirm peripheral TCL, enlarged right inguinal lymph node was excised for biopsy. Biopsy specimen of inguinal lymph node revealed effacement of nodal architecture by an atypical lymphoid infiltrate. The infiltrates consisted of medium to large sized lymphoid cells. The atypical cells showed hyperchromatic nuclei and discernible cytoplasm (Figure 2(d)). Some atypical cells had open chromatin and nuclear convolution, but nuclear pleomorphism was not evident. These atypical cells were intermixed with small reactive lymphoid cells and plasma cells. The atypical cells were positive for CD3, indicating T cell lymphoma. PCR analysis demonstrated T cell receptor gene rearrangement. EBV was not detected on PCR analysis. She received 3 cycles of chemotherapy with etoposide containing regimen. PTCL was regressed partially after 3 cycles of chemotherapy, but she refused to receive more chemotherapy. She died of disease progression 6 months later.

## 3. Discussion

There are several explanations for the development of HL and subsequent TCL such as therapy induced, immunodeficiency related, and tumor biological relations [1, 3, 6]. TCL occurring about 2 years after chemotherapy of HL and showing more aggressive clinical courses might be considered to be treatment induced [3, 6, 7]. The persistent immune dysregulations are known to be associated with HL [8]. Brown et al. described a case of HL and anaplastic large cell lymphoma (ALCL) in which a faint T cell receptor (TCR) gene rearrangement was detected in the initial HL. It suggested that this rearrangement had arisen from an oligoclonal population of reactive T cells because the RS cells were positive for CD20 [3]. The initial and persistent oligoclonal T cell expansion might have permitted the emergence of the ALCL. Thus, the ALCL and possibly the HL developed as a result of a persistently abnormal immune microenvironment. The development of HL and B cell lymphoma would be explained by clonal progression of malignant B cells through mutational accumulation and progression into a more aggressive, higher grade B cell lymphoma, because neoplastic RS cells are a type of B lymphocytes in most cases [9]. The pathogenesis of cases of classic HL and TCL is more difficult to explain. HL is rarely of T cell lineage, probably fewer than 5% of all cases [4]. Two recent studies focused on RS cells that express aberrant T cell antigens and found that only 15% to 20% of these harbor clonal TCR gene rearrangements [10, 11]. Davis et al. reported a case in which lymphomatoid papulosis, HL, and cutaneous TCL, occurring during a period of 14 years, were derived from a single T cell clone, as determined by PCR [11]. Therefore, it seems likely that HL and TCL derive from the same precursor, similar to that of HL and B cell lymphoma. Sanchez et al. reported a case of composite HL and TCL associated with Epstein Barr virus infection and suggested the possibility of malignant transformation of a preexisting T cell population developed in response to a neoplastic EBV-positive RS cells [9]. Our patient was diagnosed with peripheral TCL 2 years after the diagnosis of HL. RS cells of our case revealed no TCR gene rearrangement and EBV on PCR. Therefore, the occurrence of TCL in this case is probably related to chemotherapy. However, we are not sure whether composite lymphoma existed when HL was diagnosed, because we obtained just only supraclavicular lymph node and confirmed HL. When she received chemotherapy for HL, she suffered from diarrhea and was diagnosed with chronic nonspecific colitis with focal erosion by colonoscopic biopsy. Colonoscopic biopsy that was performed 2 years after diagnosis of HL revealed TCL, also supporting sequential development of HL and TCL rather than composite lymphoma. With these results taken into account, the direct clonal relationship between HL and PTCL was observed in few reports. So, the majority of PTCL following treatment of HL could result from therapy induced immunodeficiency rather than from clonal progression.

We report a case of HL and T cell lymphoma developing in the same patient. Although it is rare, synchronous or metachronous HL and NHL can occur more often than we expect. Therefore, we may need to consider reconfirming tissue when lymphoma is not regressed enough by chemotherapy. And, the further study for pathogenesis will be required to understand the relationship of HL and NHL.
